# Metal oxides carbon xerogel nanocomposite for methanol oxidation fuel cell

**DOI:** 10.1038/s41598-025-85579-x

**Published:** 2025-02-07

**Authors:** Fatma Mohamed, Mohamed Shaban, Omnia M. Salem

**Affiliations:** 1 Chemistry Department, Faculty of Science, Beni-Suef University, Beni-Suef, 62514 Egypt; 2 Nanophotonics and Applications (NPA) Lab, Faculty of Science, Beni-Suef University, Beni-Suef, 62514 Egypt; 3 Materials Science Research Lab, Chemistry Department, Faculty of Science, Beni-Suef University, Beni-Suef, 62514 Egypt; 4 Department of Physics, Faculty of Science, Islamic University of Madinah, P. O. Box: 170, 42351 Madinah, Saudi Arabia

**Keywords:** CX, Banana peels, Methanol oxidation, Fuel cell, Theoretical approach, Chemistry, Materials science

## Abstract

The primary requirement for electrode materials in direct methanol fuel cells (DMFC) is efficient electrocatalyst that exhibit high tolerance to methanol oxidation, excellent stability, and reasonable cost. The combination of distinct active materials with distinctive architectures may facilitate the attainment of this objective. The present study included the preparation of a Carbon Xerogel Doped with various metal oxides derived from Banana peels. The nanocomposites were thoroughly examined utilizing several characterization modalities including XRD, FTIR, and SEM. The electrocatalytic performance of Carbon xerogel doped with Iron (Fe3O4/CX), carbon xerogel doped with magnesium (MgO/CX), and carbon xerogel doped with Copper (CuO/CX) about the Methanol Oxidation Reaction (MOR) was investigated using electrochemical methods such as cyclic voltammetry, impedance spectroscopy, and chronoamperometry. The results showed that the Fe3O4/CX, MgO/CX, and CuO/CX are effective electrocatalysts with an onset potential of around 1.00 V and current densities of approximately 42.98 mA cm − 2, 28.2784 mA.cm − 2, and 6.60698 mA.cm − 2, respectively, in the optimized electrolyte for methanol oxidation. The stability of Fe3O4/CX, MgO/CX, and CuO/CX electrodes was examined using chronoamperometry and the Cyclic Stability method. The results revealed that the (Fe3O4/CX) electrode exhibited outstanding stability throughout the whole 60-minute chronoamperometry Technique and demonstrated great stability for 100 cycles in the Cyclic Stability technique. The remarkable electrochemical activity and stability may be attributed to the synergistic effect of Fe3O4/CX, which provided sufficient active sites for methanol electro-oxidation and reduced the equivalent series resistance, as shown by the electrochemical impedance spectroscopy analysis. This work used environmentally friendly materials, which presents a novel opportunity to enhance the efficiency of methanol oxidation via the utilization of affordable catalysts. This study of the theoretical technique methods for establishing the route of methanol decomposition, and systematizes their confirmation with experimental data, within the methodological framework.

**Supplementary Information:**

The online version contains supplementary material available at 10.1038/s41598-025-85579-x.

## Introduction

The growing need for renewable energy as a replacement for fossil fuels has resulted in a major increase in both academic and industrial activities in recent years. These initiatives are aimed at developing sustainable technology that will attain carbon neutrality. Because of its high power density, long-term stability, and enhanced safety features, supercapacitors have been highlighted as one of the most promising energy storage device^1^.

Fuel cells, which create electricity by electrochemical energy conversion, are critical to tackling the global energy dilemma. Various types of fuel cells are being developed, with an emphasis on alcohol fuel cells due to their low operating temperatures and cost-effectiveness^2^. Methanol, ethanol, glycerol, glucose, and sugar alcohols are being investigated as potential fuel cell possibilities, with methanol standing out due to its simple chemical structure. The use of ecologically acceptable and sustainable energy supplies, such as hydrogen and carbon biofuels (methanol and ethanol), offers enormous promise for fuel cells. Because of their great efficiency and dependability, these resources are appropriate for a wide range of applications, from handheld devices to large-scale power plants^3^. The emphasis on the development and optimization of this technology is thus justified. Direct Methanol Fuel Cells (DMFCs), a form of proton-exchange membrane fuel cell (PEMFC), have a small footprint, run on liquid fuel, have a low operating temperature, and have a high energy density. These qualities make DMFCs a tempting alternative to regular batteries^4^. However, heavy platinum loading into electrodes, the expensive cost of noble metals, low durability, and sluggish kinetics in both anodic and cathodic processes all pose difficulties to the commercialization of DMFCs. Overcoming these obstacles is critical for the successful integration of DMFCs into real-world applications^5^.

Fuel cells have three parts: cathode, anode, and membrane, and each part contains its distinct purpose. Fuel cells have been viewed as a viable renewable energy source with different uses in portable electronics such as cellular phones, laptops, notebooks, battery chargers, electronic appliances, stationary objects, electric vehicles, military equipment and remote controlled gadgets^6^. These can be additionally employed in the domestic, industrial, and commercial purpose for the generation of power and heat. Fuel cell technology can also address the challenges of power fluctuation and intermittent nature associated to the other green renewable energy sources including solar and wind systems^3^.

The fundamental principle of DMFCs is based on the electrochemical reactions of methanol and oxygen in anode and cathode, respectively, demonstrated as followed equ 1,2and 3^7^:

Anode

1 $$\:{CH}_{3}OH+{H}_{2}O\to\:{CO}_{2}+{6H}^{+}+6{\text{e}}^{-},$$

Cathode

2 $${\text{3}}/{\text{2O2}}\,+\,{\text{6H}}\,+\,+\,{\text{6}}{{\text{e}}^ - } \to ~{\text{3H2O,}}$$

Overall:

3 $$\:{CH}_{3}OH+{3/2O}_{2}\:\to\:{CO}_{2}+\:{2H}_{2}O.$$

The anode must display specified features, such as good stability, a high surface area, enhanced electrical conductivity, and a significant number of electrochemically active sites^8^. For instance, the employment of porous materials with extraordinary surface areas results in an increased number of active sites, leading to the saturation of the catalyst surface with by-products created during the methanol oxidation process^9^.

Carbon xerogels have garnered considerable attention in the literature over the past decade and can be made in numerous forms (as powder, thin-film, cylinders, spheres, discs, or can be custom shaped)^10^. The most essential qualities of these materials are their high porosity, surface area and pore volume, regulated pore structure, narrow and controlled pore size distribution, low electrical resistance, and superior thermal and mechanical capabilities^11^.Carbon Xerogel is a carbon support with three-dimensional morphology that exhibits outstanding properties such a high degree of porosity, a huge surface area, controlled pore size, and good conductivity^12^. Due to its flexible surface area and porosity depending on the synthesis circumstances, carbon gel has recently found uses in diverse sector such as energy storage and catalysis^12^. Till date, most of the preparations of carbon gels were carried out in basic media utilizing sodium carbonate as a polymerization catalyst. Carbon gel affords additionally the option to be doped with metals to promote its activity^13^.

Recently, Transition metal oxides that show potential as alternative catalyst supports for DMFCs. This is due to their excellent chemical stability and capacity to tolerate higher levels of CO, which are two important criteria that affect the operation of the cell^14^. So DMFCs that use metal oxides catalysts such as NiOx, CoOx, CuOx, CrOx, ZnOx, MnOx, MgOx and FeOx, have demonstrated enhanced reaction kinetics as well as high antipoisoning potential^15^. Therefore, transition metal oxides and their composites have garnered substantial attention as electrocatalysts due to their low electrical resistance, low cost, facile synthesis, and outstanding catalytic activity^16^. the carbon xerogel generated from the Plant waste displays a large surface area, porosity, strong graphitization, and balanced hydrophilic/hydrophobicity and electronic conductivity similar to the traditional carbons, which makes it an ideal spot to add heteroatoms to improve the activity of the catalyst^12^.

With beneficial features, carbon xerogels exhibit tremendous promise in the fields of energy conversion and storage^17^. Materials derived from carbon xerogels present compelling options across various applications, serving as electrode materials for double-layer capacitors or supercapacitors, adsorption materials for gas separation, column packing materials for chromatography, and catalyst supports for hydrogen storage^10^. The catalytic performance of carbon materials is dependent on their surface characteristics, where the surface chemistry assumes a key role in affecting catalytic qualities^18^. This requires furnishing active areas capable of chemisorbing reactants and producing suitably robust surface intermediates^18^.

The current study aims to prepare carbon xerogels doped with iron, magnesium, and copper from tannins which extracted from waste plant. The new modified Carbon xerogels characterized using different modalities. Moreover, the ability of doped CXs with high electrochemical activity for methanol oxidation Reactions (MOR). The electrochemical performances of CX/metal oxides toward the MOR in a Na_2_S_2_O_8_ medium were compared. And studies IT Stability of Fe_3_O_4_/CX, MgO/CX and CuO/CX and Cyclic stability of optimum catalyst and EIS in different concentration from methanol.

## Materials and methods

### Materials

Tannin extracted from Banana Peels, NaOH (Merck Millipore), Ferric Nitrate Extra pure (LOBA CHEMIE), magnesium chloride (PIOCHEM), copper Sulphate pentahydrate ( sigma Aldrich), Nickle Chloride Hexahydrate Extra pure(LOBA CHEMIE), Cobalt (II) Chloride hexahydrate(WINLAB), polyvinyl alcohol PVA(CASTRO COMPOSITES), isopropanol(MERCK ) and distilled water .

### Methods

#### Extracted tannin from banana peels

Gather newly gathered banana peels and dehydrate them in an oven set at 60 °C for length of 24 h. Next, smash the dried banana peels into a fine powder using a grinder. Weigh out 80 g of this powdered material and submerge it in 800 milliliters of hot water, maintaining a temperature of roughly 80 °C, for a duration of approximately 2 h. Stir the mixture throughout this time. Strain the mixture through a cheesecloth or filter paper to separate the liquid (containing tannins) from the solid (finely crushed banana peel). Ultimately, apply an oven to desiccate the solution at a temperature of 105 °C for a period of 24 h in order to get solid tannin^19^.

#### Preparation of carbon xerogel doped with metal oxides

##### Carbon Xerogel doped with iron (Fe3O4/CX), carbon xerogel doped with magnesium (MgO/CX) and carbon xerogel doped with copper (CuO/CX)

Tannin extracted was dissolved in 50 ml of distilled water at room temperature. Then, 1 gram of the tannin and 1.1 ml of formaldehyde (37% w/w) were added to 9 g of (Ferric Nitrate, or Magnesium Chloride or Calcium Sulphate) to generate (Fe_3_O_4_/CX) or (MgO/CX) or (CuO/CX). Then, the solution was continually stirred. Next, 9 g of sodium hydroxide were mixed in 50 ml of distilled water and added to the mixture. Subsequently, rinsed the mixture numerous times with distilled till pH is neutral. Following this, the material was exposed to oven drying at a temperature of 100 °C for 24 h. The composites were then exposed to calcination in a muffle furnace at a specified temperature of 400 °C for 30 min in a nitrogen atmosphere^20^.

##### Preparation of CX doped with different metal oxides/graphite electrodes

For ink slurry of 0.02 g of the produced materials, i.e., Fe_3_O_4_/CX, MgO/CX or CuO/CX, with 0.01 g of polyvinyl alcohol (PVA). All the components were combined properly and dissolved in 2 ml isopropanol/distilled water 1:2. The generated mixes were poured in closed small bottle of 5 ml then sonicated for 30 min at room temperature. After that, the catalyst ink was put onto a Graphite sheet using a micropipette. The modified Graphite sheets was allowed for 24 h to dry at room temperature before the electrochemical Measurements.

#### Characterization of the catalyst

The synthesized materials’ crystalline structure was assessed through X-ray diffraction (XRD) analysis, employing an X-ray diffractometer (PANalytical, Empyrean, Netherlands) that utilized Cu Ka radiation with a wavelength of 1.54045 Å). The XRD was operated at a voltage of 40 kV and a current of 30 mA. To identify various functional groups Fourier, transform infrared (FTIR) spectra of the samples were recorded using a Vertex 70 system (Bruker, Germany) within the wavenumber range of 400–4000 cm − 1. Furthermore, the surface characteristics of the samples were examined via scanning electron microscopy (SEM) using a ZEISS Sigma 500 VP microscope. Energy-dispersive X-ray spectroscopy (STM.EDX) was used to identify the chemical composition.

#### Electrochemical measurements

The electrochemical measurements were recorded using OrigaFlex potentiostat (OrigaLys ElectroChem., OGF01A, Rillieux, France) device was used. A standard Two electrode electrochemical cell was used at room temperature. the modified Graphite Sheet with Fe_3_O_4_/CX, MgO/CX or CuO/CX was used as the working electrode and have 1 cm^2^ surface area. the counter electrode was a Pt-electrode with the same dimensions. A solution of 0.5 M Na_2_S_2_O_8_ was used as the electrolyte in the presence and absence of methanol to evaluate the electrocatalytic activity of the prepared electrodes. Cyclic voltammetry (CV) measurements were performed at scan rates 100 mV s − 1 in a potential window between − 1 and 1 V. The chronoamperometry (CA) tests were measured at 01 V for 3600 s. Electrochemical impedance spectroscopy (EIS) analysis was performed at 1 V with 10 mV amplitude in a frequency range between 100 kHz and 0.01 Hz.

#### Computations method

To fig out the change of activity of the Fe_3_O_4_/CX surface with the introduction of Co atoms, the adsorption of CH_3_OH on the Fe_3_O_4_/CX surface was investigated using the Materials Studio based on the density functional theory (DFT). The adsorption energies of CH_3_OH and CO on the surfaces this following Eq. 4

4 $$\:\varDelta\:E\:or\:{E}_{diss}={\left({E}_{adsorbate\:}\right)}_{1}-{\left({E}_{adsorbate\:}\right)}_{2}$$

where (E adsorbate)_1_ is the total energy of CH_3_OH on the Fe_3_O_4_/CX surfaces, (E adsorbate)_2_ is the total energy of CO on the Fe_3_O_4_/CX surface. Thus negative $\:\varDelta\:$E_ads_ or E_diss_ indicates exothermic chemisorption and positive values indicate an endothermic process.

## Result and discussion

### Structure properties of samples

**Fig. 1:**
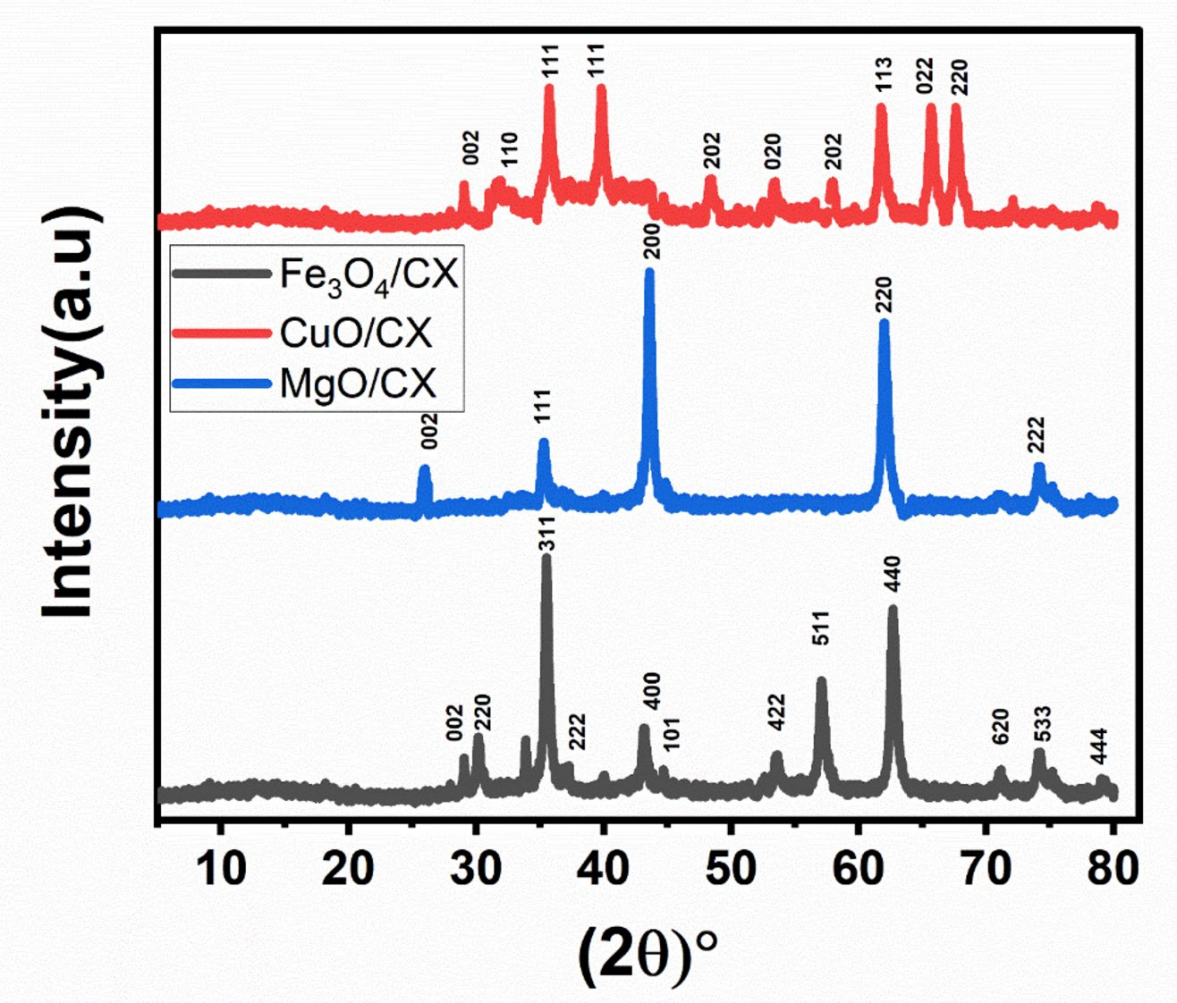
XRD of Fe_3_O_4_/CX, MgO/CX and CuO/CX.

The diffraction patterns indicated the existence of two distinct phases: graphite (JCPDS 00-012-0212) and magnetite Fe3O4 (01-089-0691). The diffractograms exhibit the characteristic carbon peaks observed at 2θ angles of 29° and 44.6°.These values approximately correspond to the (002) and (101) crystallographic planes, respectively. The graphite 002 planes displayed diffraction at a 29° angle. The integration of CX with the Fe framework has resulted in a tendency towards increased value. These modifications cause flaws in the lattice structure, leading to the displacements that are seen. The presence of this phenomenon might be attributed to the diffusion of carbon into the structure, which occurs due to the elevated temperature (400 °C) used during thermal treatment. Outside^21^.The diffraction patterns for the CX material doped with Magnesium are shown in Fig. 1. The diffractograms display distinct peaks at 2θ angles of 25.3°, 36.5°, 43.2°, 62.6°, and 75°, which are distinctive. These values closely correlate to the crystallographic planes (002), (111), (200), (220), and (222) as reported by^22^. X-ray diffraction (XRD) pattern of MgO/CX exhibits a prominent and powerful peak corresponding to the (200) orientation, indicating a high degree of crystallinity. Figure 1 illustrates the alignment and arrangement of CX doped with Copper, showcasing its orientation and crystalline structure. The recorded values of the peaks in their standing condition are as follows: 25.3°, 32.4°, 36.2°, 40°, 48.4°, 53.5°, 59.0°, 61.3°, 65°, and 67.8°. These values correspond to the crystallographic planes (002), (110), (111), (111), (202), (020), (202), (113), (022), and (220). The presence of CuO particles grouped in an orderly manner at various levels shows the successful creation of a crystalline structure^23^.

### FTIR spectra analysis

**Fig. 2:**
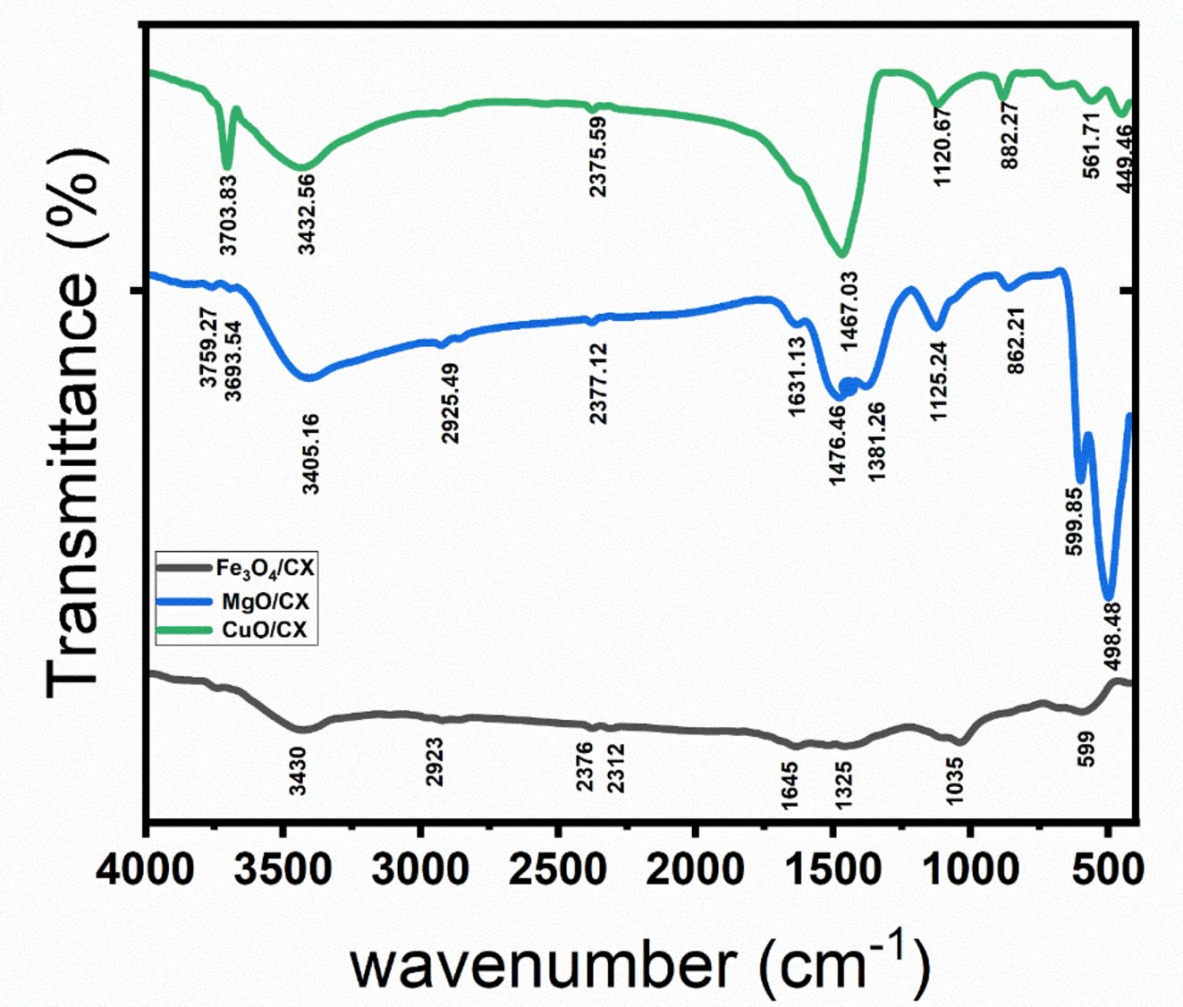
FTIR Spectra of Fe3O4/CX, CuO/CX and MgO/CX.

Figure 2 displays the infrared spectra of the adsorbents Fe_3_O_4_/CX, CuO/CX, and MgO/CX.The bands seen at 3855 cm^− 1^, 3759 cm^− 1^, 3751 cm^− 1^, and 3703 cm^− 1^ correspond to the hydroxyl (–OH) group^23^. The bands seen at wavelengths of 3430 cm ^− 1^, 3432 cm ^− 1^, 3415 cm ^− 1^, and 3405 cm^− 1^ are attributed to the hydroxyl (–OH) group, indicating the existence of O-H stretching, carboxyl, and phenolic groups^24^.The absorption peaks seen from 2925 cm^− 1^ to 2923 cm^− 1^ are due to the stretching vibration of C-H bonds. These vibrations are connected with both asymmetric and symmetric vibrations of aliphatic and aromatic structures, providing evidence for the presence of Carbon xerogel^25^. The prominent peaks at 2378 cm^− 1^, 2377 cm^− 1^, 2376 cm^− 1^, and 2375 cm^− 1^ observed in the CX material may be attributed to the presence of the C-O group, which is associated with uncompensated ambient CO_2_^24^. The absorption peaks observed at 2312 cm^− 1^ and 2310 cm^− 1^ in the case of Fe3O4/CX were attributed to the presence of − C ≡ C−^23^. The occurrence of absorption bands at 1645 cm^− 1^, 1631 cm^− 1^, and 1625 cm^− 1^ in the CX compound can be attributed to the C = C aromatic structure^26^. The bands at 1529 cm^− 1^ can be ascribed to the stretching vibrations of C = O groups in diketone, ketoester, and keto-enol structures of the Tannin precursor^26^. The peaks detected at 1476.46 cm^− 1^ and 1467.03 cm^− 1^ are associated with the –COOH functional group, and their positions were altered as a result of the introduction of MgO and CuO doping^26^. The bands within the range of 1381 cm^− 1^ to 1120 cm^− 1^, particularly for CX, were identified as the C-O vibrations associated with compounds such as esters, ethers, lactones, carboxyls, or phenols in Tannins^23^. The spectral peaks at 1035 cm^− 1^ and 1029 cm^− 1^ were identified as resulting from the stretching vibrations of C–O–C bonds, with some contributions from C–O–H and C–C–H bonds^27^. The presence of peaks at 862.21 cm^− 1^ and 882.27 cm^− 1^ can be attributed to the vibrations of alkyl amines in the C–O group’s in-plane bending and the aromatic group’s out-of-plane bending of the C–H bonds (Jiang et al., 2020). The bands observed at 599 cm-1 and 561 cm-1 correspond to the vibrations of the Fe–O bond. The Mg-O peak was seen at approximately 599.85 cm^− 1^ and 489.48 cm^− 1^, suggesting the presence of MgO^19^. The existence of peaks at wavenumbers 449.46 and 561.71 cm^–1^ verified the successful production of Cu–O^19^.

### Morphological analysis

**Fig. 3:**
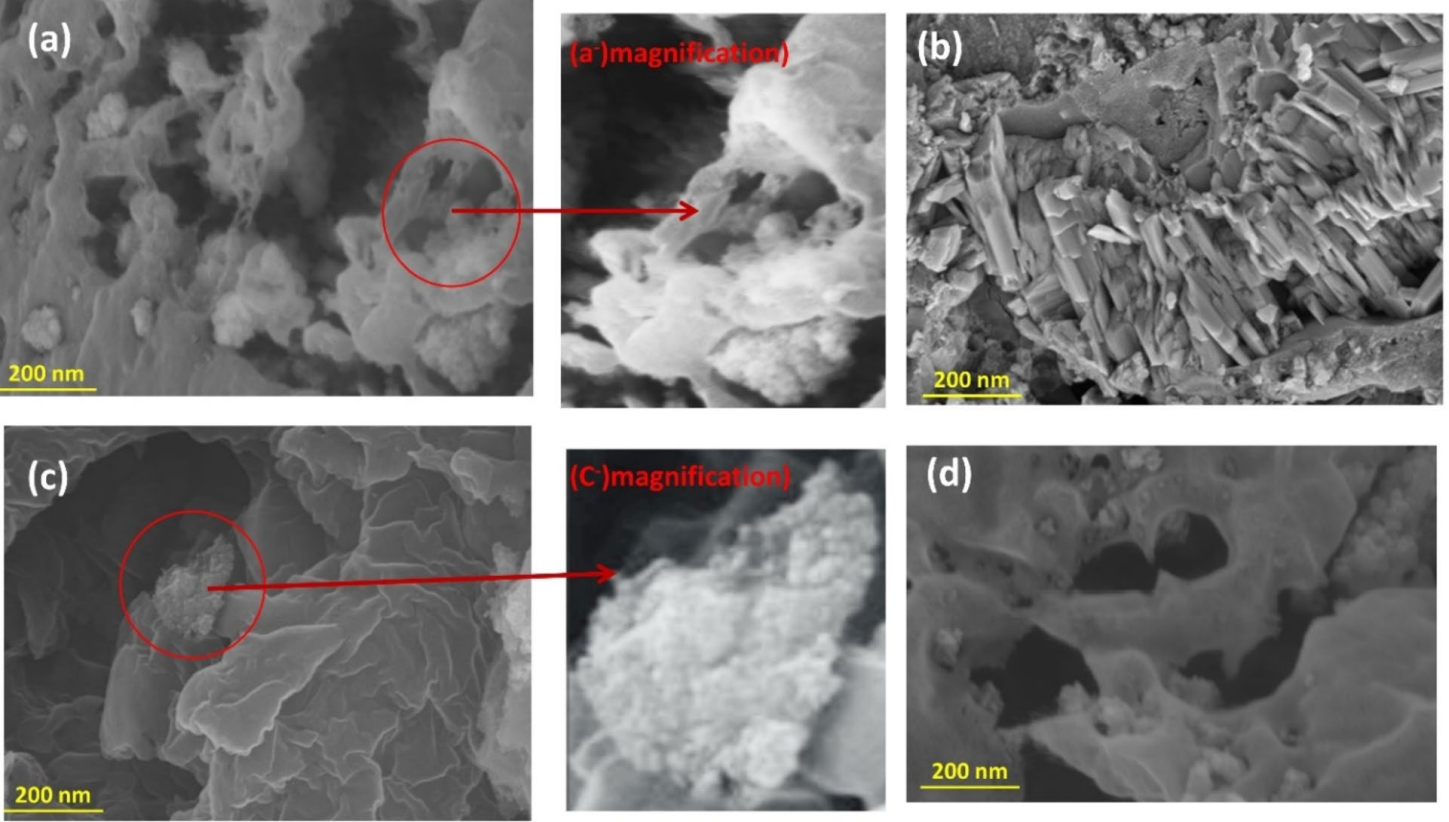
Sem of (**a**) Fe_3_O_4_/CX, (a^−^) magnification of Fe3O4/CX, (**b**) CuO/CX, (**c**) MgO/CX and (C^−^) magnification Of MgO/CX and (**d**) Fe3O4/CX after Electrochemical reaction.

Scanning electron microscopy (SEM) technique was used to study the morphology of different samples of Carbon Xerogel doped with various metal oxides. Fe_3_O_4_/CX material is composed of many layers arranged in a stacked formation. Each layer has a Fiber-like structure with varying sizes of cavities and a high level of porosity which were presented at Fig. (3). These characteristics play a crucial role in facilitating the methanol oxidation process. While CuO/CX appears as a rod-shaped morphology, as illustrated in the Fig. 3b. Furthermore, the nanohybrids efficiently protect and equally cover the material, as demonstrated in Fig. (3). The nanorod crystals exhibited a consistent and well-grown structure, appearing as one-dimensional structures. The length of these crystals ranged from 500 to 980 nm, as shown in the SEM images shown in Fig. 3b. This shape might provide drawbacks for the electrode, as it hinders quick surface reactions and blocks the access of electrolyte ions to the electrode interlayers. Consequently, this leads to a decrease in the active site and results in poor capacitance behaviour for CuO/CX. In morphology of MgO/CX indicated that Fig. (3c) demonstrate the distribution of particles in MgO/CX as clusters of irregular shaped flakes grouped as flower-like clusters. SEM reveal the crystal flakes in a particular configuration resembling a crystalline flower. The morphology of Fe_3_O_4_/CX developed may provide a suitable surface for interaction between the catalyst and methanol, thereby enhancing the methanol oxidation process. The morphology of Fe_3_O_4_/CX maintains after electrochemical measurement with presence some protrusions in the matrix as a result to consecutive measurements which respresented at Fig. 3 (d).

### Methanol oxidation fuel cell (MOFC) of Fe3O4/CX, MgO/CX and CuO/CX

**Fig. 4:**
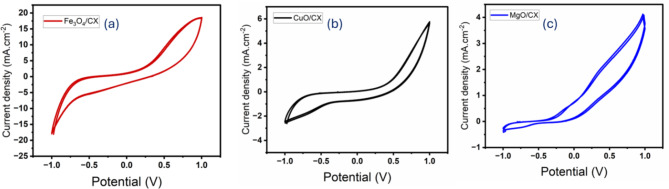
CV curves of the (**a**) Fe_3_O_4_/CX, (**b**) MgO/CX, and (**c**) CuO/CX at a fixed scan rate of 100 mV S^− 1^ in 0.5 M Na_2_S_2_O_8_.

The electro-catalytic performance of the produced electrodes Fe_3_O_4_/CX, MgO/CX, and CuO/CX was evaluated in a 0.5 M Na_2_S_2_O_8_ electrolyte solution. The potential range used was − 1 to 1 V, and the scan rate was 100 mV/s at room temperature. The results are shown in Fig. 4(a-c). The platinum wire served as the counter electrode. Figure 4(a) shows the cyclic voltammetry (CV) of the produced electrodes at a scan rate of 100 mV/s in a solution of 0.5 M Na_2_S_2_O_8_. The Fe_3_O_4_/CX electrode demonstrates superior electrochemical activity, achieving a current density of 19 mA/cm^2^. In contrast, the MgO/CX and CuO/CX electrodes exhibit lower current densities. The notable electroactivity of Fe_3_O_4_/CX can be due to the existence of a Fiber-like structure with various diameters of cavities and a high degree of porosity. This enhances the conductivity of the electrode by allowing electron transmission.

**Fig. 5:**
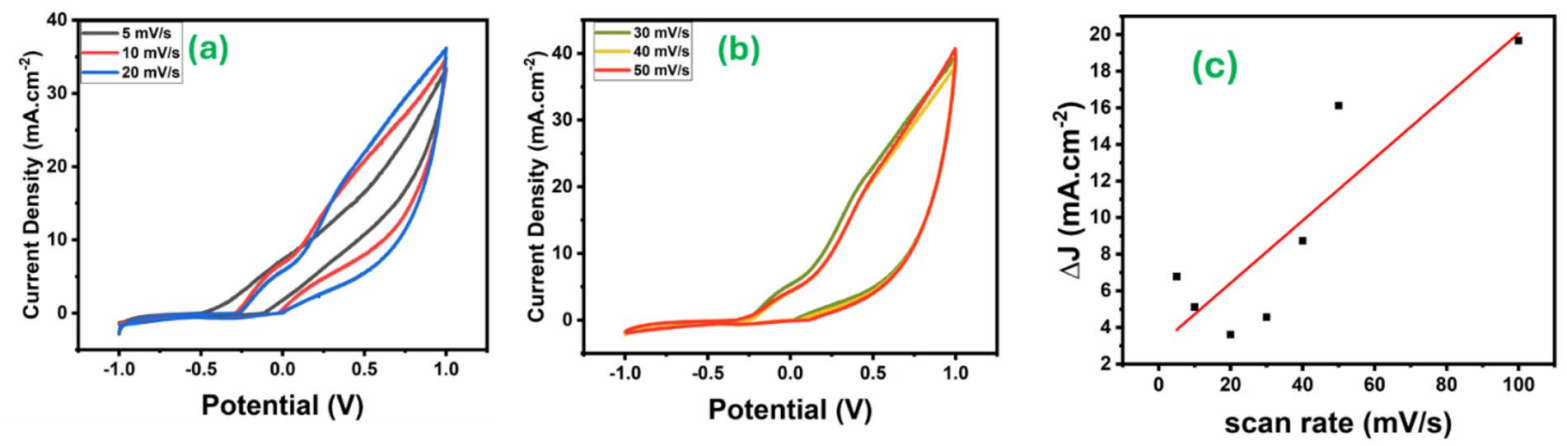
Cyclic voltametric of Fe3O4/CX at (**a**) low scan rate 5–20 mV/s, (**b**) high scan rate 30–50 mV/s in 1.5 M methanol and 0.5 M Na_2_S_2_O_8_ and (**c**) The capacitive currents (j).

The effect of varying scan rates for methanol electro-oxidation of Fe_3_O_4_/CX was studied in 0.5 M Na_2_S_2_O_8_ and 1.5 M CH_3_OH at low scan rate (5, 10, 20mV s^− 1^) and high scan rate (40,50 mV s^− 1^). The results are illustrated in Fig. 5 (a and b)., which indicates that the anodic current density gradually increases with increasing scan rate from 33.4 to 39. 3 mA.cm^− 2^ for 5 to 50 mA. S^− 1^ respectively. As the scan rate increased, the anodic peak potential (Epa) shifted towards more positive values, while the cathodic peak potential (Epc) shifted to more negative potentials. This shows that there are diffusion-controlled systems and the electro-oxidation of methanol on catalysts^24^.

The electrochemically active surface area (ECSA) of the Fe_3_O_4_/CX electrode represents the area of the electrode surface that is accessible to the electrolyte and is used for evaluating the charge transfer and/or storage. The determination of this surface area is essential in electrocatalysis to provide surface-normalized intrinsic catalytic activity. Under a non-Faradaic region, a series of CV scans were performed at different scan rates (5, 10, 20, 30, 40,50 mV s^− 1^). The average difference between the anodic and cathodic charging current densities were plotted as a function of scan rate For calculating the double-layer capacitance (Cdl)in fig() is (0.0852 Mf/CM2) and calculated The specific capacitance (Cs) is (6.908676304 mF/g) can be calculated by equations to determine ECSA.

$$\:Cdl=\:\frac{\Delta{J}}{2V} = SLOPE$$

Where ν is the scan rate in V/s, and △J are different between the anodic and cathodic current densities^25^.

$$\:{C}_{S}=\:\frac{A}{2 \times m \times v \times dV}$$

Where A: Area under the current (I) vs. potential (E) curve, m: Mass of the electrode material (mg). ν: Scan rate (mV/s). dV: Potential window (the difference in potential mV)^25^.

The ECSA for this composite electrode was found to be 12.33 cm2 illustrated in eq(). By gwyddion software determined Roughness from the SEM image is 26.33. This indicates that the ECSA when the Roughness factor is increased and which proves the effect of Fe3O4/CX on the surface area and increasing active sites^26^.

$$\:\text{E}\text{C}\text{S}\text{A}\:=\:\frac{Cdl}{{C}_{S}},$$

**Table:** 

Catalyst	ECSA (cm^2^ )	RF
Fe3O4/CX electrode	12.33	26.33

**Fig. 6:**
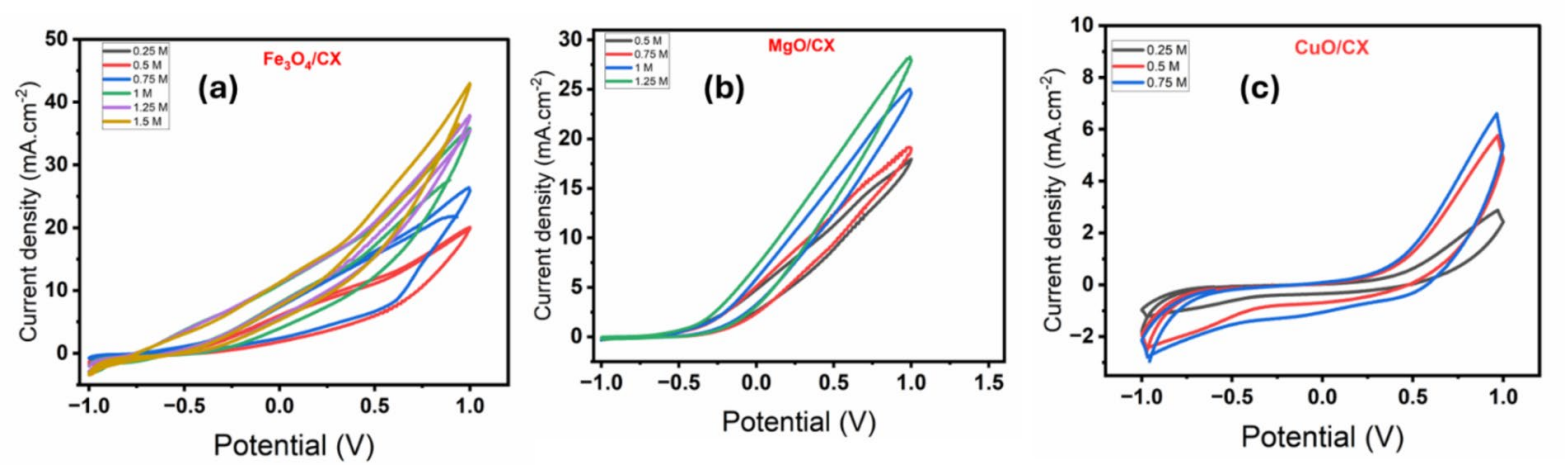
CV curves of the (**a**) Fe_3_O_4_/CX, (**b**) MgO/CX, and (**c**) CuO/CX at a fixed scan rate of 100 mV s − 1 in 0.5 M Na_2_S_2_O_8_ and with various concentrations of methanol.

To study the effect of methanol concentration on the MOR process of Fe_3_O_4_/CX, MgO/CX, and CuO/CX, the CV analysis was performed at a constant concentration of 0.5 M Na_2_S_2_O_8_ with different concentrations of methanol (0.25, 0.5, 0.75, 1, 1.25, and 1.5 M) at a scan rate of 100 mV/s which were shown at Fig. 6( a-c). When methanol concentration is increased from 0.25 M to 1.5 M, 0.25 M to 1.25 M, and 0.25 M to 0.75 M, the current density increases to 42.9862 mA.cm2, 28.3 mA.cm^− 2^, and 6.6 mA.cm2 during the MOR in Fe_3_O_4_/CX, MgO/CX, and CuO/CX, respectively. However, this trend is reversed when the methanol concentration reaches 2 M, 1.5 M, and 1 M in Fe_3_O_4_/CX, MgO/CX, and CuO/CX, respectively. A further decrease in current density is observed. The reason probably can be the resistance against the occupancy of active sites of the catalyst with the side-products resulting from the oxidation of methanol after a certain critical concentration^27^.did not improve the final activity and even prevented molecule diffusion^28^. Therefore, This behavior indicated that the MOR depended on the available active sites for methanol adsorption and the oxidation reaction was a diffusion-controlled process^29^. The methanol concentration of 1.5 M of Fe_3_O_4_/CX, 1.25 M of MgO/CX and 0.75 M of CuO/CX were chosen as the optimum concentration to be used in the Salts solution.

**Fig. 7:**
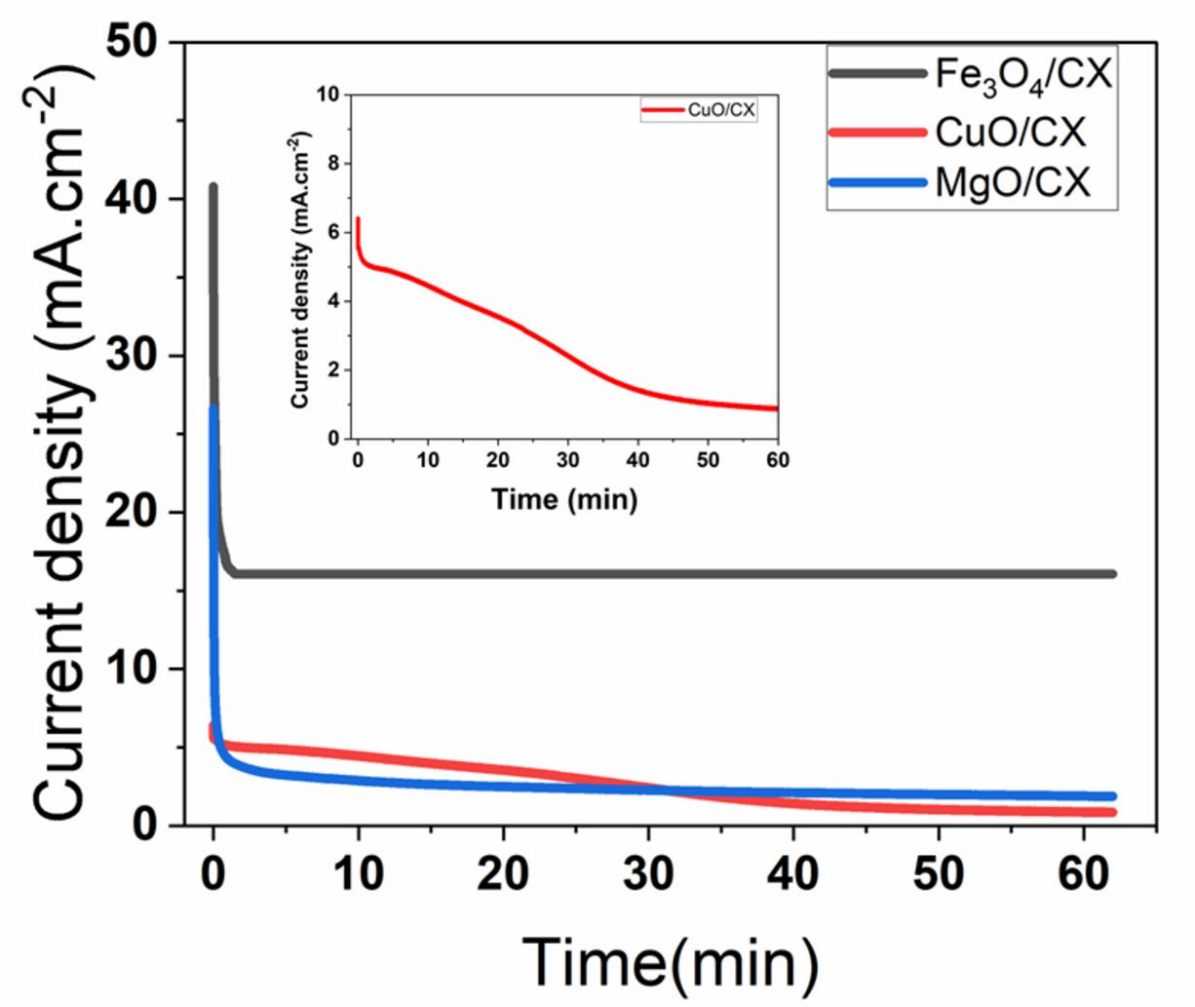
Chronoamperometric stability curves for Fe_3_O_4_/CX in 1.5 M methanol, MgO/CX in 1.25 M methanol and CuO/CX in 0.75 M methanol at room temperature at in 0.5 M Na_2_S_2_O_8_.

A chronoamperometric test was carried out to characterize the stability of the methanol oxidation reaction for the Fe_3_O_4_/CX, MgO/CX and CuO/CX in 0.5 M Na_2_S_2_O_8_ and 1.5 M, 1.25 M and 0.75 M of methanol respectively at room temperature for 3600s at Fig. 7. The results showed that the peak current values of the catalyst decreased rapidly in the early stage of measurement from 40 mA.cm^− 2^ to 17 mA.cm^− 2^ For Fe_3_O_4_/CX, 26.6 mA.cm^− 2^ to 5 mA.cm^− 2^ For MgO/CX and For CuO/CX This showed a slow decrease until the end of measurement to 16 mA cm^− 2^, 4.9 mA. cm^− 2^ and 1.3 mA. cm^− 2^ for Fe_3_O_4_/CX, MgO/CX and CuO/CX respectively. The initial rapid decline is caused by poisoning of the electrode surface or by the adsorption of CO, hydrocarbons, and oxygenates^30^. As seen in Fig. 7 the Carbon Xerogel with Different metal oxides catalyst started with a higher peak current. It showed better stability and catalytic efficiency after 3600 s.

As a result, it has been shown that Fe_3_O_4_/CX has higher catalytic activity and efficiency and this means that the favorite catalyst used in fuel cell energy is Fe_3_O_4_/CX. Besides, Fe3O4/CX has been demonstrated to possess superior catalytic activity and efficiency, making it the preferred catalyst for fuel cell energy. The Fe3O4/CX consists of many layers organized in a stacked arrangement. The structure of each layer consists of fibers arranged in a manner similar to that of a fiber. These fibers have different sizes of empty spaces and a significant level of porosity. The design of the surface facilitates the interaction between the catalyst and methanol, leading to an enhanced process of methanol oxidation it was seen that the data obtained were comparable with MgO/CX and CuO/CX. The higher values were obtained the proposed mechanism of the methanol oxidation process can be expressed as follows using a 6 electrons mechanism in Eqs. (5), (6), (7) and (8).

5 $$\:catalyst-{CH}_{3}OH\:\to\:\:catalyst-{{CH}_{3}OH}_{ads}$$

6 $$\:\text{c}\text{a}\text{t}\text{a}\text{l}\text{y}\text{s}\text{t}-{\text{C}\text{H}}_{3}\text{O}\text{H}\:\text{a}\text{d}\text{s}\:+\:4{\text{O}\text{H}}^{-}\:\to\:\:\text{c}\text{a}\text{t}\text{a}\text{l}\text{y}\text{s}\text{t}-{\left(\text{C}\text{O}\right)}_{\text{a}\text{d}\text{s}\:}+\:4{\text{H}}_{2}\text{O}\:+\:{4\text{e}}^{-}\:\:\:\:$$

7 $$\:\text{c}\text{a}\text{t}\text{a}\text{l}\text{y}\text{s}\text{t}\:+\:{\text{O}\text{H}\:}^{-}\to\:\:\text{c}\text{a}\text{t}\text{a}\text{l}\text{y}\text{s}\text{t}-{\text{O}\text{H}\:}_{\text{a}\text{d}\text{s}}\:+\:{\text{e}}^{-}\:\:\:\:\:\:\:\:\:\:\:\:\:\:\:\:\:\:\:\:\:\:\:\:\:\:\:\:\:\:\:\:\:\:\:\:\:\:\:$$

8 $$\:\text{c}\text{a}\text{t}\text{a}\text{l}\text{y}\text{s}\text{t}-{\text{C}\text{O}}_{\text{a}\text{d}\text{s}}\:+\:\text{c}\text{a}\text{t}\text{a}\text{l}\text{y}\text{s}\text{t}-{\text{O}\text{H}\:}_{\text{a}\text{d}\text{s}}+\:{\text{O}\text{H}\:}^{-}\:\to\:\text{c}\text{a}\text{t}\text{a}\text{l}\text{y}\text{s}\text{t}\:+\:{\text{C}\text{O}}_{2}\:+\:{\text{H}}_{2}\text{O}+{\text{e}}^{-}\:\:\:\:\:$$

**Fig. 8:**
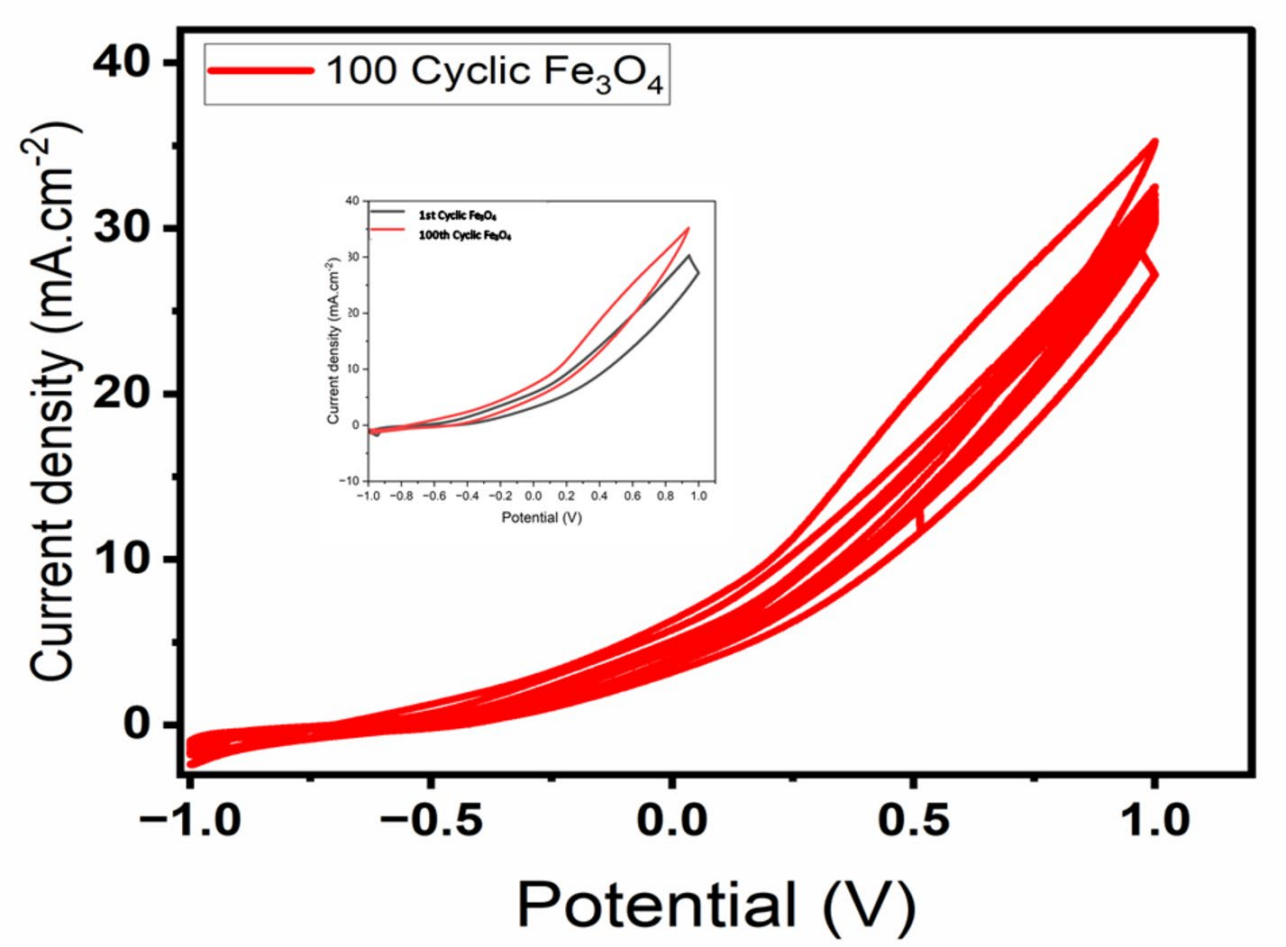
Cyclic stability of Fe_3_O_4_/CX for 100 cycles in 1.5 M methanol and 0.5 M Na_2_S_2_O_8_.

One of the crucial characteristics of a catalyst suitable for use in fuel cells is its ability to maintain cyclic stability during the process of methanol oxidation reactions (MOR)^31^.To assess the durability of Fe_3_O_4_/CX in the methanol oxidation process, a series of 100 consecutive cyclic voltammetry (CV) cycles were conducted in a salt solution containing 0.5 M Na_2_S_2_O_8_ in presence of 1.5 M methanol at room temperature, with a scan rate of 100 mV/s which presented at Fig. 8. Observably, after the 100th cycle, Fe_3_O_4_/CX retains approximately 82% from its performance, accompanied by a slight reduction in its peak oxidation current density. In the initial cycles, the electrodes interact with active sites shared with electrolyte ions and methanol. However, as the number of cycles increases, the catalyst surface becomes saturated with methanol byproducts. Consequently, there is an initial decline in current density followed by its stabilization. Additionally, the consistent stability of iso-potential points and the absence of distortion in CV curves highlight the pronounced electrochemical stability of the catalyst throughout the Methanol Oxidation Reactions process^27^. These observations signify the excellent stability of Fe_3_O_4_/CX in the context of Methanol Oxidation Reaction.

**Fig. 9:**
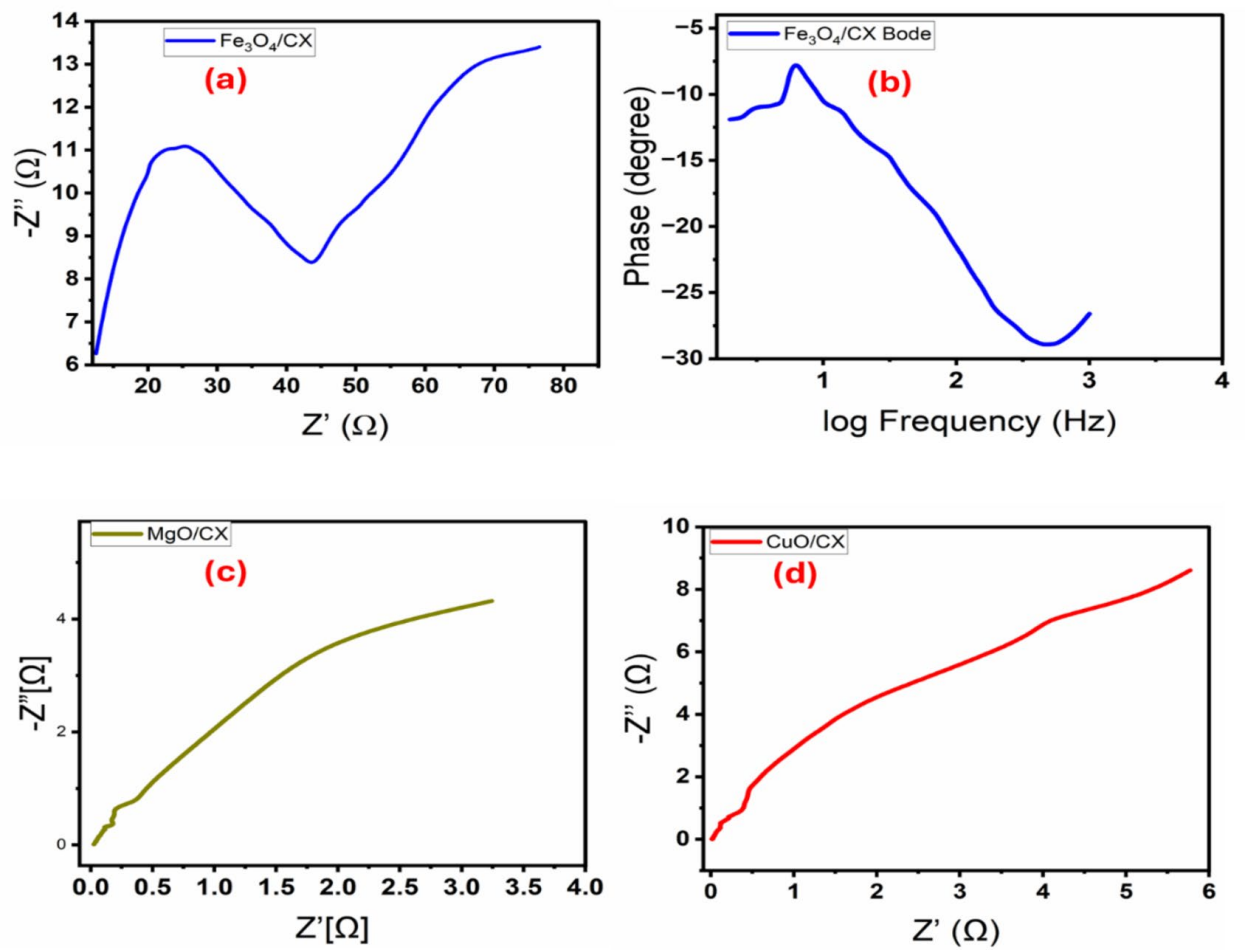
(**a**) Nyquist plot of electrochemical impedance spectrum of Fe_3_O_4_/CX measured in Na_2_S_2_O_8_ at 0 V (vs. Ag/AgCl) under illumination provided with the equivalent Randles Cell. (**b**) Bode Plot for the same cell, (**c** and **d**) Nyquist plot of electrochemical impedance spectrum of MgO/CX and CuO/CX measured in Na_2_S_2_O_8_ at 0 V (vs. Ag/AgCl) under illumination provided with the equivalent Randles Cell.

The impedance of an electrochemical system is determined by the charge transfer between the active electrode and the electrolyte contact^32^. Figure 9(a-d). displays a Nyquist plot of Fe_3_O_4_/CX, MgO/CX and CuO/CX measured in 0.5 M Na_2_S_2_O_8_ and 1.5 M ,1.25 M and 0.75 M from Methanol of Fe_3_O_4_/CX, MgO/CX and CuO/CX correspondingly Typically, two sections can be distinguished in Nyquist plots: semicircles related to charge-transfer (Rct) and capacitive (Qdl) impedances, and linear parts attributable to mass-transfer (Warburg) resistance^33^. These EIS spectra clearly demonstrated mixed kinetic and diffusion control systems^34^. The composites Fe_3_O_4_/CX, MgO/CX, and CuO/CX exhibited higher conductivity and reduced equivalent series resistance (Rct), as evidenced by the semicircles and linear patterns that were drawn. Figure 7 demonstrates that Fe_3_O_4_/CX had the lowest semicircle with a Rct of (44 KΩ), significantly less than those of MgO/CX (15 KΩ) and CuO/CX (24 KΩ), indicating that Fe_3_O_4_/CX has rapid and effective electron transport. These catalysts demonstrate the importance of the inclusion of Fe_3_O_4_ with Carbon Xerogel, which reduces the resistance of Fe_3_O_4_/CX and enhances its conductivity.

Bode graphs for the Fe_3_O_4_/CX electrode in Na_2_S_2_O_8_ in presence of 1.5 M Methanol and 25 ◦C was represented at Fig. 9b. Bode plots of Fe_3_O_4_/CX showed the fluctuation of the total impedance with frequency, inset of Fig. 7b. and the variation of phase with frequency. This figure depicts the resistive regime at low frequencies in addition to capacitive contribution at high frequencies^32^. These regimes are associated to the charge transfer resistance (Rct) and the double-layered capacitance (Cdl) of the electrode. The highest phase shift (max = -28.94 ◦) is detected at 2.65 Hz.

**Fig. 10:**
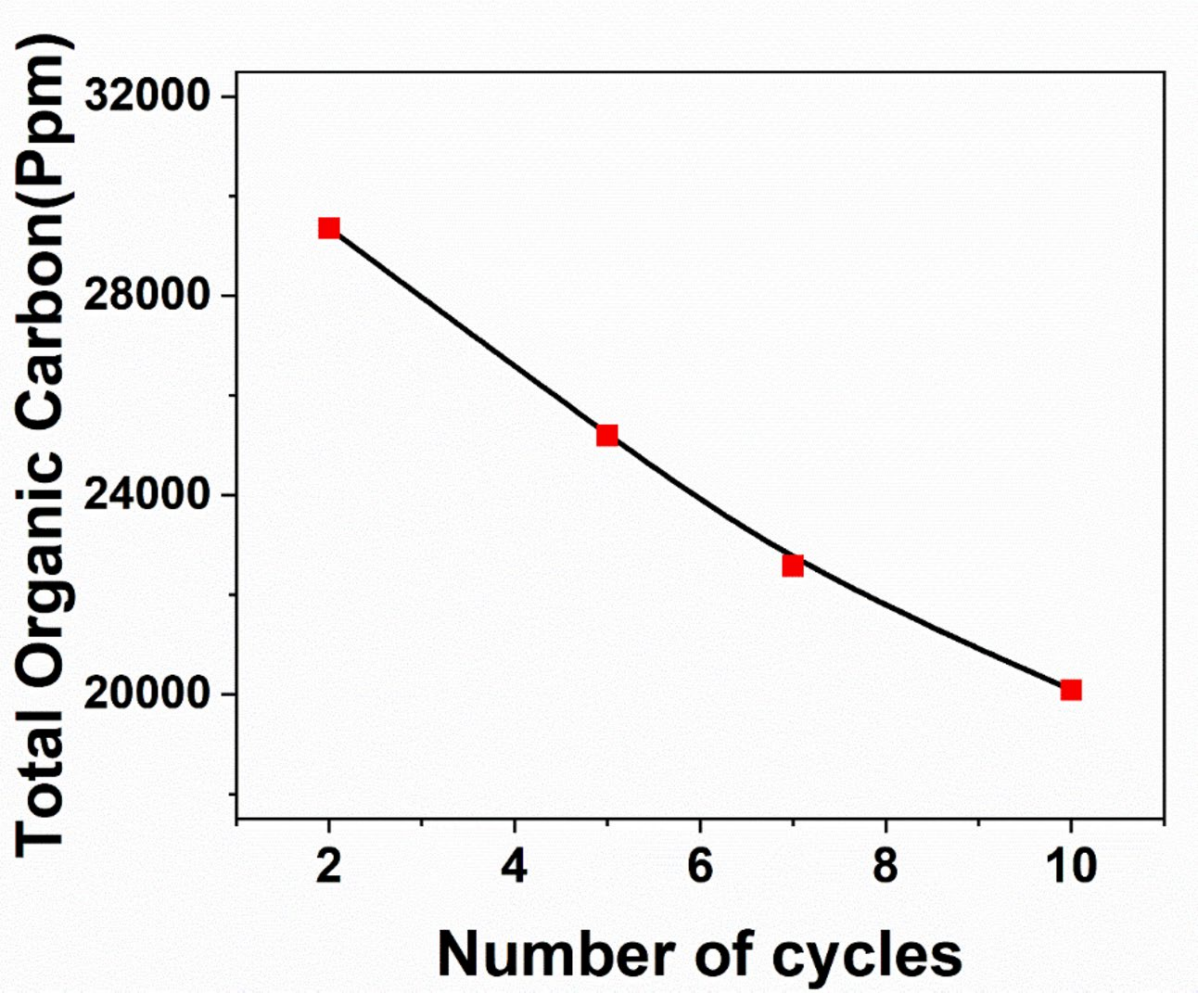
Total organic carbon (TOC) with repeated cycles over spent Fe_3_O_4_/CX at 0.5 M Na_2_S_2_O_8_, 1.5 M of methanol, and 100 mV s − 1 scan rate.

In order to verify the oxidation of methanol and reduce its concentration in the solution, measurements of total organic carbon (TOC) were conducted. In order to address this matter, the electrolyte samples were obtained from the electrolytic cell to measure the total organic carbon (TOC) over several cycles of methanol oxidation. The measurements were conducted at a scan rate of 100 mV s^− 1^, using a solution containing 0.5 M Na_2_S_2_O_8_ and 1.5 M methanol. The selected cycles for methanol oxidation were cycles 2, 5, 7, and 10. Figure 10 illustrates that the significant decrease in total organic carbon (TOC) content was caused by the oxidation of methanol on the Fe_3_O_4_/CX catalyst. This phenomenon can be attributed to the oxidative conversion of methanol into inorganic carbon, specifically CO_2_ and carbonates. The electrochemical activity of the spent Fe_3_O_4_/CX electrode leads to the oxidation of inorganic carbon, resulting in a decrease of approximately 30% in the methanol content of Na_2_S_2_O_8_ as the electrolyte^35^.

**Fig. 11:**
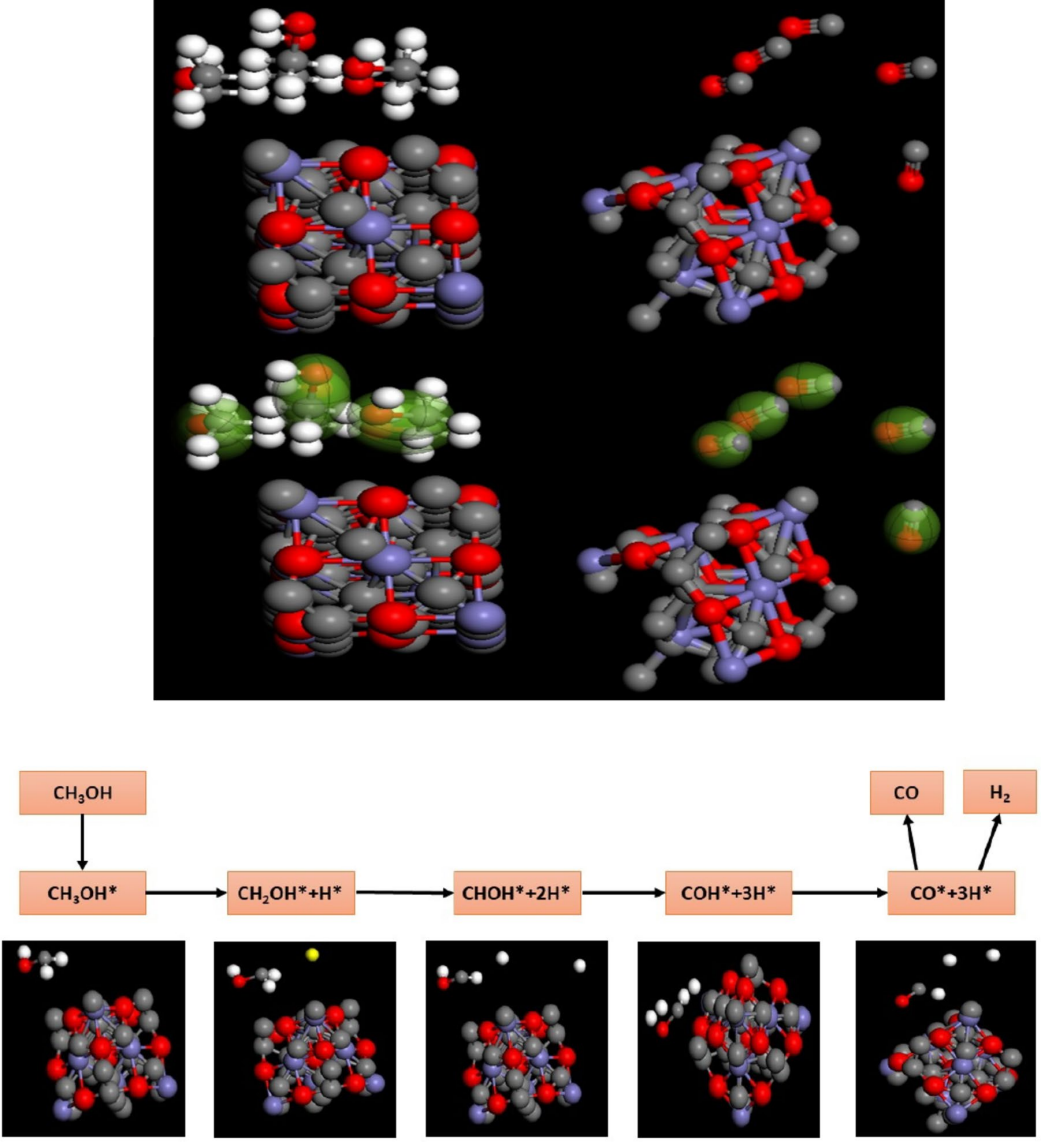
Representation of the methanol oxidation through Fe3O4/CX.

To clarify the impact of Fe_3_O_4_/CX in the methanol oxidation, the process of methanol dissociation and the ability of Fe_3_O_4_/CX to tolerate CO verified using DFT simulations. Given that the (111) facet of both electrocatalysts was mostly active for the reaction, we devised a cleaved surface for the (111) facet of the Fe_3_O_4_/CX. The process of CH_3_OH dehydrogenation initiates with the adsorption of CH_3_OH onto the electrocatalysts. Subsequently, the dissociative chemisorption of CH_3_OH occurs in a sequence of successive stages. The process leads to the creation of hydroxymethyl intermediates (CHxOH*) and surface-adsorbed protons (H*), as depicted in the figure. The optimization of atomic energy in the dehydrogenation of CH3OH leads to the formation of C-H or O-H bonds, resulting in the sequential generation of intermediates such as CH_2_OH (C-H bond cleavage), CHOH (O-H bond cleavage), and CHO (O-H bond cleavage). Prior to examining the energetic properties of CH_3_OH dehydrogenation, we performed computations to evaluate the structural modifications and binding energies of the reaction intermediates. We confirm that our adsorption energies are in good agreement with those obtained from other theories in terms of fairness and accuracy. In general, the addition of CX-doped iron facilitates a thermodynamically favorable step-by-step removal of hydrogen from CH_3_OH^36^, leading to a reduction in the energy needed for the last stages of hydrogen removal illustrated in Eq. (9). The final steps involve the reaction pathway that results in the creation of adsorbed CO and H species on Fe_3_O_4_/CX (111) with an energy of -0.215 eV shown in Eq. (10). The energy of this system is affected by the effects of Fe_3_O_4_/CX (111) on the reaction pathway with the highest level of activity which was graphed at Fig. 11.

9 $${\text{CH}}_{{\text{3}}} {\text{OH }} \to {\text{ CH}}_{{\text{3}}} {\text{O }} \to {\text{ CH}}_{{\text{2}}} {\text{O }} \to {\text{ CHO }} \to {\text{ CO}},$$

$${\text{E}}_{{{\text{diss}}}} = {\text{ E}}_{{{\text{ad}}}} \left( {{\text{CH}}_{{\text{3}}} {\text{OH}} - {\text{Fe}}_{{\text{3}}} {\text{O}}_{{\text{4}}} /{\text{CX}}} \right){\text{ }} - {\text{ E}}_{{{\text{ad}}}} \left( {{\text{CO}} - {\text{Fe}}_{{\text{3}}} {\text{O}}_{{\text{4}}} /{\text{CX}}} \right) ,$$

10 $${{\text{E}}_{{\text{diss}}}}={\text{ }} - 0.{{733ev }}--{\text{ }}\left( { - 0.{{518 ev}}} \right)\,=\, - \,0.{{215ev}}.$$

In addition to the experimental examination, DFT simulations were performed to gain understanding of the electrical influence of CO inclusion on the Fe_3_O_4_/CX surface. The DFT experiments revealed that the adsorption energies of methanol (E_ads_) on Fe3O4/CX (111) and its facets were − 0.733 eV. The energy required for the dissociation of methanol and its conversion to carbon monoxide is -0.215 eV. The negative ∆E_ads_ signifies an exothermic chemisorption process.

## Conclusion

This study presents the fabrication of nanocomposites involving of CX and metal oxides (Fe_3_O_4_, CuO, and MgO) using a straightforward method that does not require annealing or energy-intensive procedures. The nanocomposites are synthesized by utilizing tannin extracted from banana peels. The F_3_O_4_/CX material consists of many layers that are placed in a stacked configuration. The structure of each layer is similar to the fibres with different sizes of empty spaces and a significant level of porosity. This allows for the smooth flow of methanol oxidation process. The oxidation and dissociation using F_3_O_4_/CX was confirmed via Density Functional Theory (DFT).Furthermore, Fe_3_O_4_/CX demonstrated exceptional catalytic efficiency in the oxidation of methanol in a direct methanol fuel cell .It also maintains a retention of 82% and possesses the lowest charge transfer resistance of 44 Ω compared to the other produced electrodes. Hence, our study advocates for the uncomplicated procedure of producing nanocomposites consisting of CX-metal oxides, with the intended purpose of using them as anode components in DMFCs.

## Electronic supplementary material

Below is the link to the electronic supplementary material.

Supplementary Material 1

## Data Availability

All data generated or analysed during this study are included in this published article.

## References

[1] Tong, Y., Yan, X., Liang, J. & Dou, S. X. Metal-based electrocatalysts for methanol Electro-Oxidation: Progress, opportunities, and challenges. *Small* **17** (9). 10.1002/SMLL.201904126 (2021). 10.1002/smll.201904126 31608601

[2] Rashidi, S. et al. “Progress and challenges on the thermal management of electrochemical energy conversion and storage technologies: Fuel cells, electrolysers, and supercapacitors.* Progress in Energy and Combustion Science*, vol. 88. 10.1016/j.pecs.2021.100966 (Elsevier Ltd, 2022).

[3] Baruah, B. & Deb, P. Performance and application of carbon-based electrocatalysts in direct methanol fuel cell.* Mater. Adv.*** 2**(16), 5344–5364. 10.1039/d1ma00503k (2021).

[4] Forootan Fard, H., Khodaverdi, M., Pourfayaz, F. & Ahmadi, M. H. Application of N-doped carbon nanotube-supported Pt-Ru as electrocatalyst layer in passive direct methanol fuel cell. *Int. J. Hydrogen Energy* **45** (46), 25307–25316. 10.1016/j.ijhydene.2020.06.254 (2020).

[5] Tellez-Cruz, M. M., Escorihuela, J., Solorza-Feria, O. & Compañ, V. Proton exchange membrane fuel cells (Pemfcs): Advances and challenges.* Polymers*** 13**(18). 10.3390/polym13183064 (2021). 10.3390/polym13183064 PMC8468942 34577965

[6] Arshad, M. S. et al. Advances and perspectives on solid oxide fuel cells: from nanotechnology to power electronics devices.* Energy Technol.*** 11**(12). 10.1002/ente.202300452 (2023).

[7] Xia, Z., Zhang, X., Sun, H., Wang, S. & Sun, G. Recent advances in multi-scale design and construction of materials for direct methanol fuel cells.* Nano Energy*** 65**. 10.1016/j.nanoen.2019.104048 (2019).

[8] Ugalde-Reyes, O., Liu, H. B., Roquero, P., Alvarez-Ramirez, J. & Sosa-Hernández, E. EIS and relaxation times study for CO adsorbed on bimetallic Pt-Mo catalysts during the methanol oxidation reaction. *Electrochim. Acta* **418**10.1016/j.electacta.2022.140309 (2022).

[9] Salarizadeh, P., Taghi, M., Moghadam, T. & Askari, M. B. Comparison of methanol oxidation reaction process for NiCo 2 O 4 /X (X = rGO, MWCNTs, HCNs) nanocatalyst. 10.1016/j.diamond.2022.109534 (2022).

[10] Mokary Yazdely, T., Ghorbanloo, M. & Hosseini-Monfared, H. Oxovanadium catalyst based on nano-porous carbon xerogel: an efficient heterogeneous nano-catalyst for aerobic oxidation of olefins. *Desalin. Water Treat.* **158**, 256–265. 10.5004/dwt.2019.24226 (2019).

[11] Jin, H. et al. Enhanced electrocatalytic performance of N-doped carbon xerogels obtained through dual nitrogen doping for the oxygen reduction reaction. *RSC Adv.* **12** (21), 13440–13447. 10.1039/d2ra01238c (2022). 35520134 10.1039/d2ra01238c PMC9067370

[12] Mansy, H. E., Khedr, M. H. & Abdelwahab, A. Palladium functionalized carbon xerogel nanocomposite for oxygen reduction reaction. *Mater. Chem. Phys.* **313**. 10.1016/j.matchemphys.2023.128797 (2024).

[13] Bailón-García, E., Drwal, E., Grzybek, T., Henriques, C. & Filipa Ribeiro, M. Catalysts based on carbon xerogels with high catalytic activity for the reduction of NOx at low temperatures. 10.1016/j.cattod.2020.03.004 (2020).

[14] Hsu, Y. H., Nguyen, A. T., Chiu, Y. H., Li, J. M. & Hsu, Y. J. Au-decorated GaOOH nanorods enhanced the performance of direct methanol fuel cells under light illumination. *Appl. Catal. B Environ.* **185**, 133–140. 10.1016/j.apcatb.2015.11.049 (2016).

[15] Liaqat, R. et al. Fabrication of metal (Cu and Cr) incorporated nickel oxide films for electrochemical oxidation of methanol. *Crystals* **11** (11). 10.3390/cryst11111398 (2021).

[16] ur Rehman, A. et al. Fabrication of binary metal doped CuO nanocatalyst and their application for the industrial effluents treatment.* Ceram. Int.*** 47**(5), 929–5937. 10.1016/j.ceramint.2020.11.064 (2021).

[17] Gommes, C. J. & Chaltin, F. The electrical impedance of carbon xerogel hierarchical electrodes. *Electrochim. Acta* **433**, 141203. 10.1016/j.electacta.2022.141203 (2022).

[18] Ding, D., Yang, S., Qian, X., Chen, L. & Cai, T. Nitrogen-doping positively whilst sulfur-doping negatively affect the catalytic activity of biochar for the degradation of organic contaminant. 10.1016/j.apcatb.2019.118348 (2019).

[19] Kilpeläinen, P., Liski, E. & Saranpää, P. Optimising and scaling up hot water extraction of tannins from Norway spruce and scots pine bark. *Ind. Crops Prod.* **192**, 116089. 10.1016/J.INDCROP.2022.116089 (2023).

[20] de Moraes, N. P. et al. Synthesis of novel ZnO/carbon xerogel composites: Effect of carbon content and calcination temperature on their structural and photocatalytic properties. *Ceram. Int.* **45** (3), 3657–3667. 10.1016/J.CERAMINT.2018.11.027 (2019).

[21] de Moraes, N. P. et al. Zinc oxide/carbon xerogel composites for photocatalytic applications developed through acidic and alkaline synthesis routes: structural, morphological, and photocatalytic evaluations. *J. Nanopart. Res.* **22** (6). 10.1007/s11051-020-04893-9 (2020).

[22] Nigam, A., Saini, S., Rai, A. K. & Pawar, S. J. Structural, optical, cytotoxicity, and antimicrobial properties of MgO, ZnO and MgO/ZnO nanocomposite for biomedical applications. *Ceram. Int.* **47** (14), 19515–19525. 10.1016/j.ceramint.2021.03.289 (2021).

[23] Siddiqui, V. U., Ansari, A., Chauhan, R. & Siddiqi, W. A. Green synthesis of copper oxide (CuO) nanoparticles by Punica granatum peel extract.* Materials Today: Proceedings*, 751–755 (Elsevier Ltd, 2019). 10.1016/j.matpr.2020.05.504.

[24] Boostani, N., Vardak, S., Amini, R. & Mohammadifard, Z. Optimization of Ni-Co-metallic-glass powder (Ni60Cr10Ta10P16B4) (MGP) nanocomposite coatings for direct methanol fuel cell (DMFC) applications. *Int. J. Hydrogen Energy* **48** (27), 10002–10015. 10.1016/j.ijhydene.2021.10.010 (2023).

[25] Raveendran, A. et al. Layer-by-Layer Assembly of CTAB-rGO-Modified MXene Hybrid films as multifunctional electrodes for hydrogen evolution and oxygen evolution reactions, supercapacitors, and DMFC applications. *ACS Omega* **8** (38), 34768–34786. 10.1021/acsomega.3c03827 (2023). 37780023 10.1021/acsomega.3c03827 PMC10536025

[26] Salarizadeh, P., Askari, M. B. & Di Bartolomeo, A. MoS2/Ni3S2/reduced graphene oxide nanostructure as an electrocatalyst for alcohol fuel cells. *ACS Appl. Nano Mater.* **5** (3), 3361–3373. 10.1021/acsanm.1c03946 (2022).

[27] Askari, M. B. et al. Hierarchical nanostructures of MgCo2O4 on reduced graphene oxide as a high-performance catalyst for methanol electro-oxidation. *Ceram. Int.* **47** (11), 16079–16085. 10.1016/j.ceramint.2021.02.182 (2021).

[28] Askari, M. B. et al. MnCo2O4/NiCo2O4/rGO as a catalyst based on binary transition metal oxide for the methanol oxidation reaction. *Nanomaterials* **12** (22). 10.3390/nano12224072 (2022). 10.3390/nano12224072 PMC9694504 36432357

[29] Kotp, A. A., Abdelwahab, A., Farghali, A. A., Rouby, W. M. A. E. & Enaiet Allah, A. Evaluating the electrocatalytic activity of flower-like Co-MOF/CNT nanocomposites for methanol oxidation in basic electrolytes. *RSC Adv.* **13** (40), 27934–27945. 10.1039/d3ra05105f (2023). 37736558 10.1039/d3ra05105f PMC10509782

[30] Bekmezci, M. et al. Synthesis of a functionalized carbon supported platinum-iridium nanoparticle catalyst by the rapid chemical reduction method for the anodic reaction of direct methanol fuel cells. *New. J. Chem.* **46** (45), 21591–21598. 10.1039/d2nj03209k (2022).

[31] Elancheziyan, M. et al. Facile synthesis of polyaniline/titanium carbide (MXene) nanosheets/palladium nanocomposite for efficient electrocatalytic oxidation of methanol for fuel cell application. *Fuel* **303**10.1016/j.fuel.2021.121329 (2021).

[32] Mohamed, F., Rabia, M. & Shaban, M. Synthesis and characterization of biogenic iron oxides of different nanomorphologies from pomegranate peels for efficient solar hydrogen production. *J. Mater. Res. Technol.* **9** (3), 4255–4271. 10.1016/j.jmrt.2020.02.052 (2020).

[33] Abbasi, Y., Jalali, F. & Sheikhi, S. Preparation of nickel/copper sulfides from metal-organic frameworks. Applications to energy storage in a symmetric supercapacitor and electrocatalytic methanol oxidation. *J. Alloys Compd.* **938**. 10.1016/j.jallcom.2022.168450 (2023).

[34] Ying, D. et al. Insight into pseudocapacitive-diffusion mixed kinetics and conversion-alloying hybrid mechanisms of low-cost Zn-Mn perovskite fluorides anodes for powerful Li-ion/dual-ion storage. *Chem. Eng. J.* **388**. 10.1016/j.cej.2020.124154 (2020).

[35] Ruiz-López, E., Caravaca, A., Vernoux, P., Dorado, F. & de Lucas-Consuegra, A. Over-faradaic hydrogen production in methanol electrolysis cells. *Chem. Eng. J.* **396**. 10.1016/j.cej.2020.125217 (2020).

[36] Moura, A. S., Fajín, J. L. C., Mandado, M. & Cordeiro, M. N. D. S. Ruthenium-platinum catalysts and direct methanol fuel cells (DMFC): A review of theoretical and experimental breakthroughs.* Catalysts*** 7**(2). 10.3390/catal7020047 (2017).

