# Supplementary material for: Metal oxides carbon xerogel nanocomposite for methanol oxidation fuel cell
Source: Sci Rep. 2025 Feb 7;15:4603. doi: 10.1038/s41598-025-85579-x (PMC11805975; doi:10.1038/s41598-025-85579-x)
Supplement: Supplementary file 1 — Supplementary Material 1 [file 41598_2025_85579_MOESM1_ESM.docx]

**Supplementary information**

**
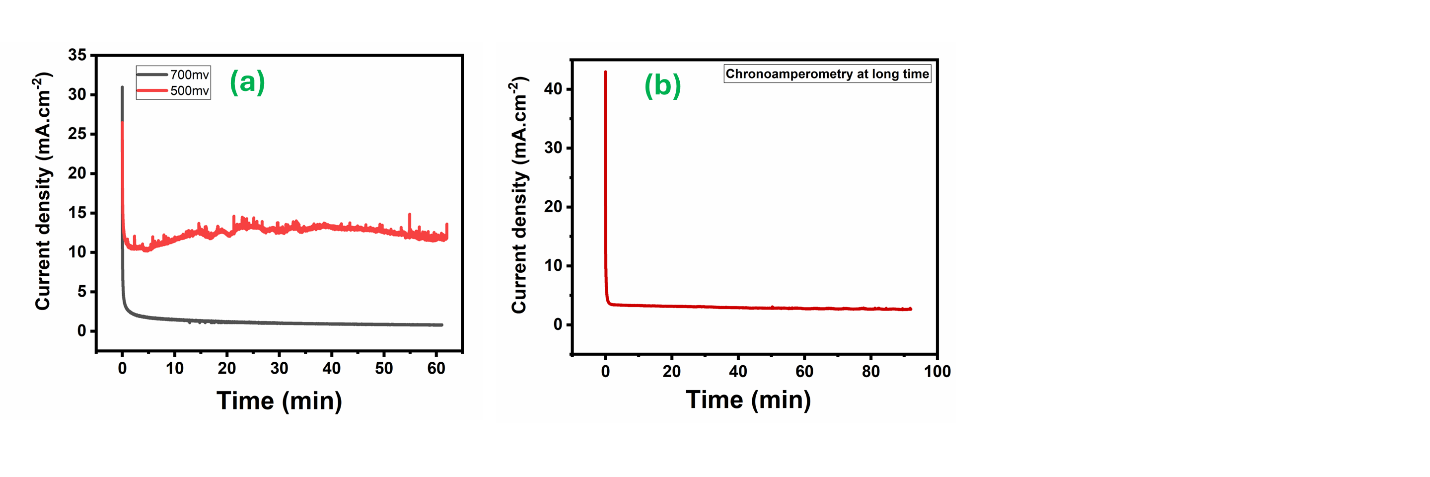
**

**Fig S1 (a) chronoamperometric stability curves for Fe_3_O_4_/CX in 500mV and 700 mV at 1.5Mmethanol (b) (a) chronoamperometric stability curves for Fe_3_O_4_/CX in 1000mV and**
